# Implementation of the 2006 Convention on the Rights of Persons with Disabilities in Zimbabwe: A review

**DOI:** 10.4102/ajod.v7i0.389

**Published:** 2018-10-22

**Authors:** Cowen Dziva, Munatsi Shoko, Ellen F. Zvobgo

**Affiliations:** 1 Nehanda Centre for Gender and Cultural Studies, Great Zimbabwe University, Zimbabwe

## Abstract

**Background:**

The Convention on the Rights of Persons with Disabilities came into place in 2006, as the main instrument for advancing the human rights of persons with disabilities. For many African states, the Convention came amidst ubiquitous marginalisation and discrimination of persons with disabilities. As expected, the Convention has been hailed as a landmark in the struggle to reframe the needs and concerns of persons with disabilities.

**Objectives:**

This article reviews the implementation of the Convention by the Zimbabwean government.

**Method:**

The study relies on reviews of extant literature on disability rights. Reviewed documents include the Convention, constitution and other related national laws, policies and measures pertaining to disability rights.

**Results:**

This article lauds the state for promulgating a disability-friendly constitution that resembles the Convention to effectuate a human rights approach to disability issues. Relatedly, the state came up with institutions that collaborate with research institutes and disability organisations to conduct research, provide services to persons with disabilities, raise awareness and advocacy and litigate for disability rights.

**Conclusion:**

In spite of these efforts, this article shows that Zimbabwe has yet to close the gap on the ideals of the Convention, mainly because of limited resources amongst state-funded institutions for advancing disability issues. The government of Zimbabwe is challenged to domesticate all provisions of the Convention and to provide resources to institutions for progressive realisation of the rights of persons with disabilities.

## Introduction and background

On 13 August 2006, the United Nations General Assembly adopted the Convention on the Rights of Persons with Disabilities (CRPD) and its Optional Protocol to promote and protect the rights of people with disabilities (PWDs) (UN Enable 2006). The framework entered into force on 03 May 2008, after receiving its 20th ratification (UN Enable 2006). The CRPD was adopted after continuous participation by various stakeholders – state and civil society as well as PWDs themselves. For this reason the CRPD is regarded as the first treaty that provides for an ensemble approach to the protection and promotion of the rights of an estimated 15% of the world’s population living with varied forms of disability (WHO & World Bank 2011).

The majority of PWDs are found in the Global South, where they experience exclusion, vulnerability to abuse and violence, lack of access to health services, employment, education, income, social support and civic involvement (Mandipa & Manyatera 2014; WHO & World Bank 2011) and are more likely to experience multiple deprivations as compared to their non-disabled peers (Mitra et al. 2014).

The CRPD confirms a paradigm shift from viewing PWDs as recipients of charity to bearers of human rights and partners for achieving sustainable development. In essence, the CRPD plays a dual role: as a development and a human rights protection instrument. The human rights role of the CRPD is reiterated by the preamble, which underscores that PWDs must fully enjoy all human rights and fundamental freedoms on an equal basis with others (CRPD 2016). The CRPD further defines ‘disability’ in a broad and inclusive manner, which indicates a model shift in approaches to disability. For this reason the CRPD:

… constitutes a shift from traditional ways of looking at disability as individual impairment to focusing on State obligations to dismantle a *disabling* environment and, in its stead, create an *enabling* environment which is inclusive and accommodates all human beings in their diversity. (Ngwena et al 2013:vii)

Thus, the CRPD is a comprehensive and well–thought-out framework with clauses meant to deal with the main challenges of PWDs. The components of the CRPD include the introductory part (articles 1 to 9), which defines terms and explains the purpose of the Convention. Specific political, social, economic and cultural rights of PWDs are explained in detail in articles 10–30. This part is followed with implementation and monitoring mechanisms (articles 31–40). The Convention ends with articles 41–50, which govern the operations of the CRPD. A closer look at this synopsis shows the extent to which the CRPD recognises, reaffirms or guarantees existing rights in the Universal Declaration of Human Rights of 1948. By so doing, the CRPD did not create a new set of rights for PWDs but rather confirmed that PWDs are human beings equal to others, as their rights under the CRPD are the same as other instruments. As such, the ideals of the CRPD become compelling norms to be implemented by every state. Principles of humanity are indeed fundamental and must be respected, promoted and fulfilled by every state actor and non-state actor in the Global South.

For the aforementioned reasons, the CRPD came as a beacon of hope for millions of PWDs in Africa, who are the most stigmatised, poorest and least educated citizens (Shome & Tataryn 2008). This explains why the CRPD received overwhelming support both during conceptualisation and ratification in the African continent. The initial working group that developed the framework included delegates from seven African countries: Morroco, Mali, Uganda, Cameroon, South Africa, Comoros and Sierra Leone (Lord & Stein 2013; UN Enable 2006). Likewise, 16 African countries signed the Convention on 30 March 2007, the first day the CRPD opened up for signature, and 34 African countries ratified the treaty, putting it into force (Lord & Stein 2013; UN Enable 2006).

Zimbabwe ratified the CRPD and its Optional Protocol on 23 September 2013 (Mandipa & Manyatera 2014). Although this was more than 5 years after the Convention had entered into force, the move confirmed the country’s commitment to recognise and advance the rights of PWDs. As a state party to the CRPD, Zimbabwe assumed the obligations to domesticate, promote, protect and enforce the rights of PWDs. As good as the vision of the CRPD seems to Zimbabwe, its directives require actions that go beyond mere ratification to effective implementation. That is the only way the Convention can become a progressive framework to transform societies and bring an end to rampant discrimination and violation of PWDs’ rights. Ngwena et al. (2013) noted that the CRPD creates a new vision of disability and inclusive equality, which must find its expression not merely in policy and law-making but through effective implementation. In concurrence with such an assertion Meekosha and Soldatic (2011) stated:

While the CRPD is a critical standard setting instrument for upholding disability rights, neither its signing nor ratification by nation states is sufficient to ensure substantial or rapid change. Indeed, the efficacy of any international treaty that a nation ratifies lies in domestication and devising of innovative ways for implementation at local level. (p. 1384)

It remains to be seen how Zimbabwe has fared with the obligation to implement the CRPD’s provisions for progressive realisation of PWDs’ rights. Accordingly, this article:

- takes stock of the implementation of the CRPD in Zimbabwe, focusing on how the 2013 Constitution domesticated the CRPD and the best practices by state institutions to implement provisions of the CRPD,
- unearths challenges faced by state actors in implementing provisions of the CRPD,
- proffers recommendations for action by state actors toward effective implementation of the CRPD in Zimbabwe.

The article is based on a literature review of the CRPD, the constitution of Zimbabwe, journal articles and reports on disability and development in Zimbabwe. The article comprises four sections, including the Introduction and background. This is followed with a review of the constitutional reform and how it domesticated the CRPD. The third section comprises a review of efforts to implement the CRPD by national institutions. The last section concludes the discussion and presents recommendations for action in Zimbabwe.

## Constitutional reform and implementation of the Convention on the Rights of Persons with Disabilities

In 2013, Zimbabwe promulgated a new constitution to replace the 1979 Lancaster Constitution. Whilst the 1979 Constitution condemned discrimination against PWDs, it only recognised physical disability, to the express exclusion of all other forms of disability affecting people in society. Unlike the Lancaster Constitution, the 2013 Constitution includes disability as one of the grounds prohibited for discrimination under section 56. Although the provisions of the new constitution are an improvement, in part, the new constitution follows in the footsteps of the old constitution by deploring the discrimination of persons with physical and mental disabilities whilst leaving out persons with intellectual and sensory disabilities. There is therefore a need for Zimbabwe to adopt the meaning of disability as proffered by the CRPD. However, by enacting the new constitution, the government of Zimbabwe has in part implemented the provisions of the CRPD. That is so because, under article 4(a), the CRPD directs the adoption of appropriate legislative measures for the implementation of the rights recognised in the CRPD, and in article 4(b) it directs state parties to take all appropriate measures to modify or abolish existing laws that discriminate against PWDs.

The constitution further recognises the dignity, equality and rights of all human beings, including PWDs, under its founding provisions. The constitution thus confers the duty of every human being to respect the rights of everyone, including PWDs. According to Mandipa (2013), the recognition of inherent dignity and equal worth of all human beings is especially crucial for persons with sensory (especially those with albinism), mental and intellectual disabilities, who endure being viewed as inferior human beings in society. Indeed, the recognition of inherent dignity and equal worth of all human beings in the constitution of Zimbabwe reflects the general principles found under article 3 of the CRPD.

The constitution (2013) under section 22(4) calls for agencies to take measures to ensure accessibility by PWDs to all buildings, environments and transportation to which other members of the public have access. By so doing, the constitution implements articles 9 and 19 of the CRPD (2006), calling for states to ensure that PWDs participate fully in community life and also live independently. Environmental accessibility addresses the challenges faced by PWDs in moving around and living independent lives in society. Most public and private structures are not accessible to PWDs in Zimbabwe (Mandipa 2013). This may hinder the participation of PWDs in public life, including their employment. Whilst inaccessible infrastructure may contribute to hindering PWDs from securing employment in government and private companies, it may not be the paramount reason for the unemployment of PWDs. Zimbabwe is a low income country that is experiencing economic difficulties, which have resulted in very minimal functioning of the industry and an unemployment rate of over 90% (ZimStats & UNICEF 2014). It is, however, important that a mandatory clause be included in local government laws to ensure the issuance of a certificate of completion to public and private structures after satisfying the accessibility of the structure by PWDs.

The constitution (2013) under section 22(3)(c) encourages ‘… the use and development of forms of communication suitable for persons with physical or mental disabilities’. This is supported by section 62 of the constitution, which guarantees access to information for all human beings. The inclusion of sections 22(3) and 62 implements articles 4(1)(h) and 9 of the CRPD, which encourages accessible information for PWDs in society. Whilst this proclamation by the constitution is commendable, section 62 is criticised for being silent on how persons with visual and hearing impairments can exercise this right (Mtetwa 2012).

Another important constitutional clause for ensuring access to information is section 16. This clause makes sign language one of the sixteen official languages for communication in Zimbabwe. The inclusion of sign language by the constitution is commendable, as Zimbabwe’s policy regarding official languages always excluded a huge portion of persons with hearing and speech disabilities (Mugumbate 2016). This section answers the calls by article 9(1)(b) of the CRPD for state parties to ensure access to information and communications. The inclusion of sign language in the constitution is likely to contribute towards the development of this mode of communication, thereby striving for meaningful inclusion and participation of persons with speech and hearing impairments in society (Mandipa 2013). As Hurskainen (2002) noted, language is an emblem that switches individuals from misery to plenty, from backwardness to progress and from backwaters to the centre of life. Whilst the inclusion of this right remains important for its development, resource constraints remain a real threat to realisation of this noble idea. In the judiciary circles, access to justice in courts is still compromised for persons with hearing disabilities because of a lack of sign-language interpreters (Lord & Stein 2013; Mandipa & Manyatera 2014).

The constitution, under section 83, provides for elaborate rights of PWDs. Section 83 directs the state to advance PWDs’ issues by coming up with measures:

- to enable them to become self-reliant,
- to enable them to live with their families and participate in social, creative or recreational activities,
- to protect them from all forms of exploitation and abuse,
- to give them access to medical, psychological and functional treatment,
- to provide special educational facilities for their education,
- to provide state-funded education and training where they need it.

The inclusion of PWDs’ rights under the Bill of Rights in Zimbabwe’s constitution is a positive step towards showing the importance accorded to PWDs in society. The move resembles the commitment by Zimbabwe to address some of the challenges of PWDs in socio-economic participation. PWDs are excluded in matters of concern to them in society owing to inadequate assistive devices and inaccessible structures and environments. Section 83 further speaks to the main challenges of PWDs, who often face widespread discrimination, exploitation, violence, maltreatment, limited access to health and employment opportunities, and unequal access to credit and other productive resources to become self-reliant. PWDs, and women in particular, are at an increased risk of experiencing violence, as they depend heavily on well-wishers and family members for survival and personal assistance as a result of limited educational and employment opportunities in scenarios where those who appear to assist may turn out to be assailants. Thus, the inclusion of section 83 largely embodies a constitutional commitment to articles 16, 24 and 25 of the CRPD, which address PWDs’ access to health facilities and empower them to be self-reliant so as to escape exploitation and abuse. Above all, the inclusion of PWDs’ rights under the Bill of Rights in Zimbabwe’s constitution strengthens accountability and ensures that PWDs have access to remedies, which is a fundamental concept of human rights law.

In including section 83, the constitution was mindful of the fact that PWDs face challenges to enjoy the right to education. In guaranteeing state-funded education, the constitution addresses the challenges of many children with disabilities dropping out of school because of inability to pay fees (Moyo & Manyatera 2014). Access to education for PWDs advocated by the constitution remains fundamental for students with disabilities, as it is both a human right in itself and an indispensable means for the realisation of other rights. Various studies on education concur that education is a gateway to a better future, as it increases prospects for better employment opportunities and ultimately improves life outcomes (Moyo & Manyatera 2014).

Whilst section 83 of the constitution resembles the CRPD in protecting and advancing the welfare of PWDs, some clauses within the same section are not exhaustive and lack conceptual clarity. There exists a clause in section 83 (rights of PWD) that contradicts the spirit of the CRPD for adequate resources and commitment to advancement of issues that concern PWDs. The section in question calls upon the state to come up with appropriate measures, within the limits of the resources available to it, to ensure that PWDs realise their full mental and physical potential (Constitution 2013:39). Apparently this clause only addresses the rights of PWDs and the elderly (section 82) and does not apply for other vulnerable groups such as women (section 80) and children (section 81). This clause limits the effective implementation of PWDs’ rights. Amidst serious economic challenges in Zimbabwe, it is highly expected that the clause on section 83 will become an excuse for non-implementation of the ideals of the CRPD by government agencies, citing financial problems and lack of resources.

Whereas articles 6 and 7 of the CRPD prioritise the rights of children and women with disabilities, the constitution (2013) failed to specifically provide for such groups of society. Children and women with disabilities require specific rights and protection as they face multilayered forms of discrimination. Women with disabilities face double discrimination – firstly as PWDs and secondly as women in a patriarchal society (Du Plessis 2007; Mandipa 2013; UN 2006). The plight of women with disabilities is also exacerbated by resource constraints and being powerless in society. Economic dependency and prevailing social norms continue to prevent women with disabilities from combating societal discrimination (US Embassy 2014). Regarding children with disabilities, there is persistent prejudice and discrimination against them, mostly in rural areas, because of entrenched cultural views that disability is a result of punishment from God and ancestors. Ensuing from this misconception, children with disabilities are despised and hidden from the public by their relatives to evade shame and stigma. In extreme cases, some parents strangle children with disabilities to death after birth; others sometimes hide them away when visitors arrive in fear of ridicule (Mandipa 2013). Thus, the overlooking of such engrained discrimination faced by women and children with disabilities by the drafters of the constitution leads to a contrast from articles 6 and 7 of the CRPD, which provides for specific protection for these disadvantaged groups.

The constitution resembles the CRPD by including section 120, which provides for the political representation of PWDs in the Senate. Courtesy of section 20(1)(d), two out of the 80 senatorial positions are reserved for PWDs, who are elected by PWDs through their various formations. The senators are expected to influence policy and law making that protects and takes into consideration the challenges of PWDs in society. However, the inclusion of senators with disabilities has had a limited impact in enhancing the lives of the people they represent. It is a rare case when one finds a motion moved by these senators for the plight of PWDs. Since their appointment in 2013, the senators have not done enough to lobby for a disability policy. In addition, they have failed to lobby for quick alignment of the 1992 *Disability Persons Act*, which views PWDs using the damaging medical and charity models of disability. Even with the presence of senators with disabilities, the ministry responsible for PWDs received paltry budgets in 2014 (Kachembere 2014), 2015, 2016 and 2017, just like before 2013 when Parliament had no senators with disabilities.

Similarly, section 4A of the *Urban Councils Act* (2008) implements the CRPD by allowing for the appointment of special councillors, which may bring in councillors with disabilities in local governance appointed by the minister responsible for local government. The inclusion of special needs councillors is expected to ensure adequate promotion of PWDs’ rights and welfare in local governance. Unlike the constitution, which clearly stipulates the election of two senators, there is no specific number of PWDs who should make up the 25% ceiling of special councillors to be appointed into local governance. The failure to specifically mention the number of councillors with disabilities amongst the 25% special councillors may also result in the exclusion of this disadvantaged group. Special groups are numerous, meaning the minister may select special councillors based on age, gender; linguistic, ethnic and religious grounds whilst paying no attention to PWDs. Therefore, the *Urban Councils Act* must be amended to clearly state the number of councillors with disabilities to be appointed amongst the special councillors. Also important is the amendment of the *Rural District Councils Act* to introduce special appointments for PWDs in rural councils. Adequate representation of PWDs in both rural and urban councils can go a long way to ensuring that local government policies and service delivery become sensitive to the needs of this disadvantaged group.

The CRPD under article 33(1–2) calls for the establishment of independent national institutions to advance PWDs’ issues. In line with this provision, the constitution of Zimbabwe established the Zimbabwe Human Rights Commission (ZHRC) in terms of section 242 to promote awareness and respect for human rights and freedoms of all human beings, including PWDs. The commission is empowered under section 243(k)(ii) of the constitution to visit and inspect places where PWDs are kept or stay and to inspect the human rights situation in such places. Upon its operationalisation in 2004, the ZHRC established a Special Interest Thematic Working Group in accordance with the *ZHRC Act* to help the commission in protecting, promoting and enforcing the human rights of vulnerable groups in society, including PWDs. Together with other departments within the ZHRC, the working group implements articles 8 and 31(1) of the CRPD through research on and raising awareness of PWD issues.

In 2015, the ZHRC commissioned a baseline study on the human rights situation in Zimbabwe, which revealed negative societal perceptions and attitudes towards PWDs (ZHRC Baseline Report 2015a). Together with other national institutions, the ZHRC has become an important institution for the protection and promotion of PWDs’ rights in Zimbabwe. The capacity of the commission to effectively implement the CRPD, however, remains limited by resource constraints, just like any other grant-aided institution in Zimbabwe. In 2015, just a year after its operationalisation in 2014, the ZHRC reported limited support from government and high staff turnover caused by uncompetitive remuneration and the failure to honour contractual obligations of timely remittance of wages by the end of the month (ZHRC 2015b).

## Implementation of the Convention on the Rights of Persons with Disabilities by government ministries

Various government ministries, including the Ministry of Public Service Labour and Social Welfare (MoPSLSW), have also been crucial in implementing the CRPD through ensuring that PWDs can access welfare and basic needs. This has seen the implementation of article 28 of the CRPD, which mandates that stakeholders ensure comprehensive social protection mechanisms for PWDs. In performing this role, the MoPSLSW works with research institutes, disabled person organisations, NGOs and other government ministries such as the Ministry of Primary and Secondary Education, which strives to ensure access to education for children with disabilities in line with article 24 of the CRPD, which provides for PWDs’ equal access to education. Together, the two ministries administer the Basic Education Assistance Module (BEAM), which is meant to ensure access to education for vulnerable children, including those with disabilities and those with parents with disabilities. Through BEAM, many beneficiaries realise their lifetime dream of accessing education in Zimbabwe. Important as the scheme has become, it only provides bursaries for students in special schools, as opposed to those in inclusive schools. This is promoting the sending of children with disabilities to special institutions as opposed to the inclusive education system advocated by the CRPD. Moreover, the scheme is also affected by underfunding, which results in beneficiaries sometimes being sent back home from schools because of unpaid school fees. Without adequate funding for BEAM, many children with disabilities drop out as a result of failure to raise money for fees.

Furthermore, the Ministry of Health and Child Welfare provides assistive devices to PWDs, including wheelchairs, spectacles, crutches, artificial limbs for those PWDs in need of them and treatment creams for Albinism conditions. Like other ministries, the Ministry of Health and Child Welfare is under-resourced and normally fails to adequately provide for these requirements by PWDs. As noted by Eide et al. (2006), only a quarter of PWDs who apply for assistive devices receive them, whilst the majority do not get them. Against this background, the Ministry of Health has fallen short of the standards under articles 4 and 20 of the CRPD to provide assistive aids and devices to all PWDs in need of them. Without access to the much-needed assistive devices, PWDs’ mobility and independent lives as called for by article 20 of the CRPD are compromised and schoolchildren with disabilities are likely to drop out from school.

The government of Zimbabwe also created the Office of the Special Advisor in 2007 to advise the President and cabinet on disability issues. The Special Advisor’s office is the focal point that mainstreams and implements disability-related issues within the government. Although questions have been asked regarding its mandate and appointment criterion (Mandipa 2013), the office has become a focal point for coordinating disability functions within government. Between 2013 and 2016, the office coordinated an annual National Disability Expo in a bid to provide a platform for stakeholders involved in disability issues to interact and share their experiences (Lang & Charohwa 2007; Mandipa 2013; Mandipa & Manyatera 2014). In 2016, the office in conjunction with other stakeholders brought together concerned stakeholders to share information on the relationship between health and disability. The expo provided a platform that has proved to be an avenue for advocacy and raising awareness of challenges and opportunities for implementation of article 8 of the CRPD in Zimbabwe. The first office bearer, Brigadier Felix Muchemwa, was appointed by the then-president of Zimbabwe, Robert Mugabe. Brigadier Muchemwa passed away in 2016, and the office lay idle until the new president of Zimbabwe, Emmerson Mnangagwa, appointed Joshua Teke Malinga in 2017. This was an answer to the calls by article 33 of the CRPD for states to create focal points within government for close and effective implementation of disability issues. Through lobbying and advocacy by Mr Malinga’s office, the new government of Zimbabwe endorsed the African Charter on Human and Peoples’ Rights on the rights of PWDs of January 2016 (Moyo 2018).

The government of Zimbabwe also implements the CRPD through research and documentation of disability issues. In 2013, the Ministry of Health and Child Welfare commissioned a survey in all 10 provinces of Zimbabwe entitled ‘Living Conditions among Persons with Disability’ to provide a comprehensive mapping for the lives of PWDs. Together with the national census of 2012, the studies revealed the various challenges faced by PWDs to participate in socio-economic and political development in society. However, it is concerning to note that the national census of 2012 did not bother to reveal the numbers of PWDs, their disability features and geographical location. Consequently, the available statistics on the prevalence of disability are outdated, and from past studies conducted before 2005. The failure by the Zimbabwe Statistical Agency to collect up-to-date statistics on disability issues contradicts the spirit of article 31 of the CRPD, which obligates state parties to ensure the collection of appropriate information, including statistical data about PWDs. In addition, non-prioritisation of disability issues during national censuses makes it difficult for policymakers to get information about this disadvantaged group of society and may result in their marginalisation when it comes to social protection mechanisms.

The government of Zimbabwe also works with Non-Governmental Organisations and state universities to conduct disability research to inform policy formulation and implementation as stated under articles 4(f–g) and 31 of the CRPD. State universities, including the University of Zimbabwe and Midlands State University, have established specialised departments to teach and research disability issues. In 2015, the Great Zimbabwe University also established the Jairos Jiri Centre for Special Needs Education to spearhead disability-related studies and research. The centre aspires to be the hub for transferring cutting-edge research and knowledge in special needs education. Through teaching, holding of research conferences and publication of results, the centre implements articles 4(f–g) and 31 of the CRPD.

On the other hand, Midlands State University through its faculty of law established the Disability Legal Aid Clinic in 2012 to advance disability rights. With financial support from university management and the Open Society Initiative of Southern Africa, the legal clinic aspires to become a citadel of disability advocacy and litigation through awareness raising, empirical research and publication of disability issues (MSU Website 2013). On 04 and 05 August 2016, the Legal Aid Clinic hosted the first ever clinical legal education conference to advance justice for PWDs (MSU Website 2016). Further, the Faculty of Law at Midlands State University introduced a disability rights module to equip law officers with contours in disability and law discourse (Chadenga 2014). This is in line with article 13(2) of the CRPD, which calls for effective justice for PWDs through appropriate training for administrative justice personnel, including law officers. There is no doubt that law graduates from the law school are catalysts for effective disability litigation and advocacy in society.

## Conclusion

By ratifying the CRPD, Zimbabwe committed herself to advancing PWDs’ rights and gave impetus to implementation of the provisions and to holding government accountable for compliance with the Convention. Against this background, this article reviewed the implementation of the CRPD in Zimbabwe, through evaluating the extent to which the 2013 Constitution incorporated provisions of the CRPD and the extent to which government ministries addressed the provisions of the Convention on the ground. Notably, the 2013 Constitution is an improvement from the 1979 Constitution in terms of disability rights protection. In the same human rights spirit of the CRPD, section 83 of the constitution confers human rights on PWDs like anyone else in society. To some extent the constitution strives to domesticate provisions of the CRPD and at least recognises, promotes and protects the rights of PWDs as called for by the CRPD. The study also applauds the creation of the ZHRC and the office of special advisor to the president and cabinet on disability for the purpose of advancing human rights issues, including those of PWDs. Within the limits of the resources available to these institutions, the ZHRC together with government ministries have made strides in advancing PWDs’ issues.

Although Zimbabwe has taken *de jure* steps to realise its CRPD commitments, there are major challenges in terms of realising these commitments *de facto*. Laws, policies and institutional frameworks are strong foundational instruments for realisation of PWDs rights if they are exhaustive of this disadvantaged group’s critical needs and are followed with effective implementation mechanisms. This is because of the existence of vague and weak clauses in the constitution in relation to PWDs’ rights, which is something that limits effective policy enforcement. There is also the challenge of resource constraints and the aforementioned lack of will by the government to support PWDs’ issues. As such, government and the donor community must show commitment to PWDs’ issues and fully support the cause.

This article calls for the urgent domestication of the CRPD, as well as review and alignment of all disability related laws that predate the constitution and the CRPD. Constitutional bodies and other institutions advancing the rights of PWDs should be strengthened through adequate budgets. By so doing, the institutions will be able to effectively execute their mandate and bring about change on the ground. It is clear that the CRPD places the primary obligation for implementation on state actors. Hence, the government should make efforts to strengthen coordination of these tasks and to work hand in glove with other interested players to meet the obligations of the CRPD, through awareness raising, service provision and research. The researchers of this article believe that if government works together in good spirit and faith with non-state actors, Zimbabwe will go a long way towards ensuring that the CRPD is effectively implemented to ensure that the rights of PWDs are protected, promoted and enforced.
